# A new patient-led approach to building research infrastructure and evidence generation

**DOI:** 10.1093/oxfimm/iqag009

**Published:** 2026-05-19

**Authors:** Oonagh Cousins, Harry Leeming, David Putrino, Janna Gordon

**Affiliations:** Visible Health Inc, 251 Little Falls Drive, Wilmington, DE, 19808, United States; Visible Health Inc, 251 Little Falls Drive, Wilmington, DE, 19808, United States; Cohen Center for Recovery from Complex Chronic Illness, Icahn School of Medicine at Mount Sinai, New York, NY, United States; Visible Health Inc, 251 Little Falls Drive, Wilmington, DE, 19808, United States

**Keywords:** long Covid, Myalgic Encephalomyelitis/Chronic Fatigue Syndrome (ME/CFS), patient-led research, digital health, patient and public involvement (PPI), research infrastructure

## Abstract

Over recent decades, patient and public involvement (PPI) has become a more established element of health research policy, although its implementation is often criticized for tokenism and for underrepresenting marginalized groups. In fields such as complex chronic illness (CCI), where formal research activity has historically been limited, conventional PPI frameworks have had little scope for meaningful application. Within this context, a new wave of patient-led initiatives has emerged that moves beyond participation in existing systems toward the creation of independent infrastructures for knowledge generation, extending the principle of “nothing about us, without us.” This commentary examines Visible, a patient-founded health technology platform that combines daily energy-management tools with research infrastructure for CCIs. This infrastructure enables in-house data analyses and external collaborations, including app-based data studies, investigator-led research, and integration within clinical trials. We explore the advantages of this dual-purpose model, including greater inclusivity, sustained engagement, and richer longitudinal data. We also describe how embedding research functions within tools that patients find directly useful allows evidence generation and patient support to be mutually reinforcing.

## Introduction

Complex chronic illnesses (CCIs) are diagnoses that include, but are not limited to, Long Covid, Myalgic Encephalomyelitis, Fibromyalgia and chronic tick- and vector-borne illness. CCIs are both highly prevalent and profoundly debilitating, yet those affected continue to receive very limited medical support. Despite their scale and impact, they remain among the most under-researched conditions in contemporary medicine [1–3].

Over the past three decades, patient and public involvement (PPI) has become a core component of health research, with funders and institutions increasingly recognizing the value of lived experience in shaping research priorities and outcomes [4, 5]. Evidence from multiple fields indicates that well-implemented PPI can make research more relevant and accountable to the communities it aims to serve [6].

In fields such as CCIs, where research activity itself has historically been limited, traditional PPI frameworks have had limited scope for application. In this context, some patient communities have moved beyond participation in existing systems. Rather than contributing only to studies led by external institutions, patients have begun to create independent infrastructures for knowledge generation, extending the principle of “nothing about us, without us” into the design of research systems that stand alone from traditional academic institutions [7]. These efforts reflect both the frustrations of neglect and the innovation with which patient communities are addressing persistent gaps in understanding and care.

This commentary examines the Visible platform, a patient-founded health technology initiative developed for people living with CCIs. Founded in 2022, Visible combines two functions within a single platform, operating both as a digital health tool that helps people live well with complex chronic illness, and as an infrastructure for data generation and research. This dual model reflects two central challenges in the field: the lack of effective day-to-day support and the scarcity of research evidence. Visible is presented as a case study of patient-led innovation designed to address both [8, 9].

## Introduction to patient-led research

Over recent decades, patient and public involvement (PPI) has evolved from grassroots activism to a central component of health research policy. Emerging from 1970s disability rights movements and 1980s HIV/AIDS activism, these efforts advanced the principle “nothing about us without us,” arguing that affected communities should be equal partners in knowledge generation [7, 10]. Methodologically, PPI draws from Kurt Lewin’s [11] action research and on participatory and community-based research frameworks that formalized approaches of embedding community perspectives. By the 1990s, patient advocacy had begun influencing formal research infrastructures: HIV/AIDS activism contributed to the establishment of the Patient-Centered Outcomes Research Institute (PCORI) in the US, while the UK’s INVOLVE (1996) institutionalized PPI, later becoming a National Institute for Health Research (NIHR) requirement [4].

Despite this progress, substantial critiques remain. PPI is frequently described as tokenistic [12] and as failing to adequately represent marginalized groups [13]. Traditional evidence hierarchies also continue to undervalue lived experience; for example, the widely taught “evidence pyramid” omits patient knowledge altogether [14].

Against this backdrop, new approaches have emerged. Some large research charities, such as Cancer Research UK, have sought to embed PPI systematically across the research lifecycle, moving beyond consultation toward more genuine forms of co-production [15, 16]. In parallel, a newer wave of initiatives founded and led by patients has developed independent infrastructures for advancing research, enabling communities to shape priorities and generate evidence outside traditional academic systems. Examples include the EURORDIS rare disease registries [17] and the Count Me In initiative, which allows people with cancer to contribute clinical and genomic data directly to research [18, 19].

Visible sits within this newer wave of patient-led initiatives. It demonstrates how communities affected by under-researched conditions can move beyond participation to actively shaping the systems through which evidence is generated. The platform builds on lived experience to inform its design, infrastructure, and research agenda, offering a model of patient-driven innovation in contexts where traditional research systems have been insufficient [20, 21].

## Complex chronic illness

Complex chronic illnesses (CCIs) cover a broad set of diagnoses, among them Long Covid, Myalgic Encephalomyelitis/Chronic Fatigue Syndrome (ME/CFS), Fibromyalgia, and chronic tick- and vector-borne illnesses such as Lyme disease. These conditions present significant challenges for research, as they are often not well-defined clinical entities and biomedical understanding remains limited [22, 23]. People with lived experience recognize clear overlaps and interconnections between different CCI diagnoses that existing research has not fully captured [24].

These conditions are frequently, though not exclusively, triggered by infectious events, in which case they are classified as infection-associated chronic conditions and illnesses (IACCIs) [25, 26]. Although their biological mechanisms are not yet fully understood, research has documented abnormalities across multiple physiological systems, including immune, autonomic, metabolic, and neurological domains [25, 27]. They produce debilitating, fluctuating symptoms that severely impair quality of life and participation in daily life [2, 28]. While generally non-fatal, some cases can be life-threatening [29].

Long Covid has emerged in the wake of the COVID-19 pandemic as potentially the most prevalent CCI, affecting an estimated 400 million people worldwide [1, 30, 31]. For comparison, pre-pandemic prevalence estimates for ME/CFS suggested around 71 million cases globally, or roughly 0.89% of the world’s population [32–34]. There is substantial clinical overlap between Long Covid and ME/CFS, with many individuals meeting diagnostic criteria for both conditions. As a result, the global burden of ME/CFS is likely considerably higher than pre-pandemic estimates alone would suggest [23, 25].

Beyond Long Covid and ME/CFS, millions more live with other CCIs. Across these conditions, treatment and access to appropriate care remains limited, leaving many people to manage profoundly disabling symptoms with minimal clinical support [2, 3, 35–40].

## Visible’s dual-purpose model: energy-management and research infrastructure

This section is organized as follows: first, it examines Visible’s dual-purpose function as both an energy-management tool to help users live well with CCI and research infrastructure; second, it considers the extent of its patient-led design; third, it assesses the advantages of its dual-purpose model; and finally, it considers limitations.

### Energy-tracking tool

Many people with complex chronic illness (CCI) experience symptom worsening following physical, cognitive, or emotional exertion. This includes, but is not limited to, post-exertional malaise (PEM), a hallmark of the condition ME/CFS. Pacing, the deliberate balancing of activity and rest, is recognized as playing an important role in health outcomes for those living with CCI and recommended by both the AAPM&R Long COVID Compendium and the NICE ME/CFS guideline as a core management approach, yet supportive tools remain limited within healthcare systems [2, 41].

Recent evidence indicates that heart rate (HR) metrics, including heart rate variability (HRV), may serve as useful indicators of symptom activity in CCIs [42, 43]. Resting HR measures the number of heart beats per minute, while HRV measures the time between consecutive heartbeats. Both are well-established as markers of general health in healthy populations. Although not specific to any single pathology, HRV is influenced by several interconnected systems, including cardiovascular, autonomic, respiratory, and immune, making it a valuable integrative marker of physiological function [42, 44].

Visible provides HR and HRV data to inform users in two complementary ways:

- **Real-time pacing visibility:** The wearable continuously tracks HR, using it as a proxy for exertion and physiological stress. HR data are translated into PacePoints™, a cumulative daily exertion score that supports energy budgeting across the day. When HR approaches personalized exertion thresholds, the app provides real-time notifications to prompt users to consider rest or activity adjustment.
- **Long-term pattern visibility:** Daily morning HR/HRV readings and evening symptom logs are used to support longitudinal symptom visualization and generate a morning stability score (1–5 scale), reflecting physiological stability and recovery capacity compared to one’s baseline over the last 90 days. Over time, these data allow users to identify trends in capacity, observe how different stressors affect recovery, and refine pacing strategies to support more stable day-to-day functioning.

### Research infrastructure

Alongside pacing support, Visible functions as research infrastructure through several channels:

1. In-house data science: Visible’s data science team develops algorithms to quantify and model symptom dynamics, including potential digital biomarkers, post-exertional malaise trajectories, and the role of contextual variables such as sleep quality and infection status. These analyses draw on heart rate, HRV, symptom logs, and behavioral metrics collected through the app.
2. External collaborations: Visible supports research with academic and clinical partners in three ways:
  - App-derived data analyses: Over 25,000 participants have contributed more than 437 million data points, enabling large-scale analyses of symptom dynamics and potential correlates. Examples include studies on hormonal cycles and symptom severity [45], and air quality variation and illness flare-ups [20, 21].
  - Prospective investigator-led studies: Visible has been integrated into prospective investigator-led studies exploring new methods and mechanisms in CCIs. Participants receive the app and compatible wearables as part of study protocols. Recent projects have investigated self-reported outcomes among people with ME/CFS and Long Covid, as well as the feasibility of heart-rate-guided pacing interventions [46, 47]. In these contexts, Visible is both the object of study and the research tool, functioning as a data-collection platform while also supporting participants’ day-to-day self-management.
  - Clinical trial integration: Visible has also been embedded within interventional trials as a digital endpoint and monitoring tool. It has recently been incorporated into an antiviral treatment trial [48], enabling continuous physiological monitoring and outcome assessment. Unlike many traditional trials in which participants use study-specific devices that may not provide pacing guidance or self-management support, Visible provides ongoing pacing support alongside data collection. This creates a more reciprocal model of participation, in which data generation and participation support occur simultaneously.
    In conventional CCI trials, investigators may use validated patient-reported outcome measures (PROMs) as primary or secondary endpoints. Visible does not seek to replace established clinical trial data capture systems such as REDCap or other electronic data capture (EDC) systems. However, Visible is preparing to integrate selected, validated PROMs into its platform to assist users in tracking their symptoms and functioning over time. This PROMs data would also be available for investigators to use in both retrospective and prospective analyses.
3. Recruitment facilitation: The “Trials near you” feature connects individuals with relevant research studies, based on diagnosis and location. The tool provides trial information and pre-filled enrollment forms, lowering participation barriers and expanding recruitment capacity [21].
4. N-of-1 studies: Visible supports within-person evaluations of interventions by comparing post-intervention data with historical baselines across symptom logs, HR/HRV, sleep quality, and contextual exposures. This “lab-in-the-pocket” approach enables personalized effect estimation in contexts where group-level evidence is limited [49].

### Built by people with lived experience

With more than half the Visible team directly affected by CCI including one of its co-founders, the platform’s development has been directly informed by lived experience and patient-identified needs [50]. Beyond its internal composition, Visible engages systematically with its user community to guide ongoing development through iterative design and continuous feedback processes [51].

This participatory, user-driven approach is well established in commercial technology sectors, where continuous user feedback and iterative design are central to product development. Visible has adopted these practices in several ways:

5. Founding 100 beta group: During the first six months post-launch, 100 users were invited to test and provide feedback on platform features, generating over 76 000 data points during the beta phase [52].
6. User surveys: Regular in-app surveys collect quantitative and qualitative feedback from free and paid users, guiding feature development [51].
7. Early-access feature testing: Selected user groups trial forthcoming features prior to wider release, enabling iterative refinement before general rollout [53].
8. Member support: Members can contact the team directly through the in-app Help & Feedback function, receiving replies from staff, many of whom have lived experience of CCIs [51].
9. Transparent development: Progress updates are shared publicly through blog posts and a “build in progress” campaign documenting development milestones [54].

In this way, Visible extends the principles of participatory and community-based research beyond traditional institutional settings. Lived experience is embedded not only in consultation processes, but in the design and operation of the research infrastructure itself, advancing the principle of “nothing about us, without us.”

### Significance of the dual-purpose model

Taken together, these features represent a broader conceptual development. Visible’s patient-led team recognized that progress in CCI research may be strengthened by linking evidence generation to the tools patients already use day-to-day. In other words, a dual-purpose model that functions both as a support tool and as research infrastructure.

Unlike conventional research infrastructures, which separate patient care from data collection, this approach aligns individual support with collective knowledge production. In doing so, it challenges the traditional distinctions between “research subject” and “care recipient” [55].

This dual-purpose model offers several potential advantages:

- Inclusion and accessibility: The model is particularly relevant for energy-limiting conditions, in which symptom severity and variability often restrict participation in conventional research. By embedding research functions within a home-based self-monitoring tool, Visible lowers practical barriers and may enable participation from a broader, more representative population, including those who are severely affected.
- Longitudinal data: CCIs are characterized by dynamic, fluctuating symptoms that are difficult to capture in single-timepoint studies or longitudinal studies with pre-determined assessment intervals. Visible enables continuous biometric and self-reported data collection in real-world contexts, generating longitudinal data that can capture symptom variability and flare-up trajectories over time [56]. Such data may complement conventional trial designs by providing a more detailed understanding of symptom dynamics between scheduled assessments. This approach may be particularly relevant for clinical trials in CCIs, where symptom variability and flare-up patterns are central to patient experience but not always systematically measured.
- Sustained engagement: Because the platform provides immediate utility for daily self-monitoring and activity pacing, users are more likely to remain engaged over time. This sustained engagement facilitates the generation of rich, long-duration datasets that are harder to achieve in conventional designs.
- Iterative feedback and refinement: Continuous use of the platform generates data that improves both scientific understanding and product improvement. Aggregated data inform algorithmic refinements, which in turn enhance the insights available to users for pacing decisions, creating a reciprocal relationship between research and self-monitoring.
- Sustainable funding: CCI research has historically been constrained by scarce public and private investment. Visible’s tiered-access model provides a self-sustaining source of research funding: core features are available without cost, while subscription services support the research infrastructure. This structure seeks to balance accessibility with long-term financial viability.
- Transparency and reciprocity: Visible prioritizes transparent communication. Findings are shared in accessible formats, showing how individual contributions advance collective knowledge. This reframes participation as collaboration rather than passive data provision, consistent with participatory health models [55].
- Alignment with lived experience: Traditional research priorities in the CCI field have not always reflected patient concerns. As a patient-founded initiative, Visible seeks to ensure that research questions remain grounded in lived experience, directing attention to domains historically overlooked.

### Limitations

Although Visible demonstrates the potential of patient-led innovation, several limitations should be acknowledged. First, as a direct-to-consumer platform, data collection relies on users to self-report symptoms and diagnoses. In the absence of clinical verification, there is a risk of diagnostic uncertainty and reporting inaccuracies, which may affect data quality and the performance of algorithms developed on the platform. Second, engagement with the platform requires access to digital technologies and sufficient health capacity to use them, potentially excluding some of the most marginalized or severely affected individuals. Third, while the subscription-based model provides financial independence from traditional grant funding, it also raises equity concerns. Costs are not currently covered by public healthcare systems or insurance providers, which may limit access for some groups. Fourth, although Visible follows best practices common to health technology start-ups, it does not yet operate within the formal governance frameworks that typically guide publicly funded or academic research infrastructures. Finally, the wearable devices integrated into the platform are not currently FDA-approved for medical use, meaning that the data generated cannot currently be used to support medical decision-making.

## Conclusion

Visible illustrates how patient-led initiatives in CCI can move beyond participation toward shaping the research infrastructures through which evidence is generated and shared, extending the principle of “nothing about us without us” to the level of infrastructure itself [7]. In doing so, the platform challenges the conventional separation between care and data collection, aligning individual support with collective knowledge production.

Although Visible was developed to address challenges specific to CCIs such as Long Covid and ME/CFS, its underlying principles may have broader applicability. For example, the dual-purpose model has attracted interest from communities studying cancer-related fatigue, heart failure rehabilitation, multiple sclerosis, post-concussion syndrome and other long-term conditions. Whether similar approaches prove feasible in these contexts remains to be determined, but the combination of patient-centered design, continuous real-world data generation and financial sustainability may offer a potential template for addressing evidence gaps in other areas of medicine.

Further information about the Visible platform is available at: https://www.makevisible.com/. The Visible mobile application is available for iOS and Android devices.

## Data Availability

No new data were generated or analyzed in support of this commentary. Sources cited are publicly available as referenced.

## References

[1] WHO. Clinical Management of COVID-19: Policy Brief. Geneva: WHO, 2024.

[2] NICE. *Myalgic Encephalomyelitis (or Encephalopathy)/Chronic Fatigue Syndrome: Diagnosis and Management (NG206)*. 2021. https://www.nice.org.uk/guidance/ng206 (April 2025, date last accessed). 35438859

[3] Vardaman M , Gilmour S. Time to correct the record on the global burden of Myalgic Encephalomyelitis/Chronic Fatigue Syndrome. J Transl Med 2025;23:331. 40087760 10.1186/s12967-025-06281-0 PMC11907965

[4] Palm ME, Evans D, Staniszewska S et al Public involvement in UK health and care research 1995–2020: reflections from a witness seminar. Res Involv Engagem 2024;10:65. 38909270 10.1186/s40900-024-00598-8 PMC11193893

[5] Domecq J , Prutsky G, Elraiyah T et al Patient engagement in research: A systematic review. BMC Health Serv Res 2014;14:89. 24568690 10.1186/1472-6963-14-89 PMC3938901

[6] The Lancet Rheumatology. Patient and public involvement: Better research for patients. Lancet Rheumatol 2025;7:e73. 39880486 10.1016/S2665-9913(25)00004-9

[7] Charlton JI. Nothing about us Without us: Disability Oppression and Empowerment. Berkeley, CA: University of California Press, 1998.

[8] Visible. *About | Visible*. 2025. https://www.makevisible.com/about (April 2025, date last accessed).

[9] Visible. Activity tracking for illness, not fitness. 2025. https://www.makevisible.com/ (November 2025, date last accessed).

[10] Strand DL , Holen M. Patient-led research and displacements of biomedical knowledge production, distribution, and consumption. Health 2025;29:276–94. 38676312 10.1177/13634593241249096

[11] Lewin K. Action research and minority problems. J Soc Issues 1946;2:34–46.

[12] Ocloo J , Matthews R. From tokenism to empowerment: progressing patient and public involvement in healthcare improvement. BMJ Qual Saf 2016;25:626–32. 10.1136/bmjqs-2015-004839 PMC4975844 26993640

[13] Jameson C , Haq Z, Musse S et al Inclusive approaches to involvement of community groups in health research: the co-produced CHICO guidance. Res Involv Engagem 2023;9:76. 37679854 10.1186/s40900-023-00492-9 PMC10486022

[14] Sibley M. We need to change the hierarchy of evidence-based medicine. BMJ Opinion. 16 November 2020. Available at: https://blogs.bmj.com/bmj/2020/11/16/miles-sibley-we-need-to-change-the-hierarchy-of-evidence-based-medicine/ (May 2026, date last accessed).

[15] Cancer Research UK. *Patient and Public Involvement in Research*. 2024. https://www.cancerresearchuk.org/for-researchers/how-we-deliver-research/patient-and-public-involvement-in-research (April 2026, date last accessed).

[16] Cancer Research UK. *Patient Involvement Toolkit for Researchers*. 2024. https://www.cancerresearchuk.org/patient-involvement-toolkit-for-researchers (April 2026, date last accessed).

[17] EURORDIS & NORD. *Rare Disease Patient Registries: EURORDIS–NORD–CORD Joint Declaration of 10 Key Principles*. EURORDIS, 2011. https://download2.eurordis.org/documents/pdf/EURORDIS_NORD_CORD_JointDec_Registries_FINAL.pdf (April 2026, date last accessed).

[18] Count Me In. *Empowering Patients to Accelerate Cancer Research*. 2024. https://joincountmein.org/ (April 2026, date last accessed).

[19] Broad Institute. *Count Me In.* 2024. https://www.broadinstitute.org/count-me-in (April 2026, date last accessed).

[20] Visible. *Research Update: New Study, New Insights*. 2025. https://www.makevisible.com/blog/research-update-new-insights-new-study (April 2026, date last accessed).

[21] Visible. Introducing: Clinical trials near you. 2025. https://www.makevisible.com/blog/introducing-clinical-trials-near-you (November 2025, date last accessed).

[22] Institute of Medicine. Beyond Myalgic Encephalomyelitis/Chronic Fatigue Syndrome: Redefining an Illness. Washington, DC: National Academies Press, 2015. 25695122

[23] Komaroff AL , Bateman L. Will COVID-19 lead to Myalgic Encephalomyelitis/Chronic Fatigue Syndrome? Front Med (Lausanne) 2020;7:606824. 33537329 10.3389/fmed.2020.606824 PMC7848220

[24] Davis HE , Assaf GS, McCorkell L, et al Characterizing long COVID in an international cohort: 7 months of symptoms and their impact. EClinicalMedicine. 2021;38:101019. 10.1016/j.eclinm.2021.101019 PMC8280690 34308300

[25] Davis HE , McCorkell L, Vogel JM, Topol EJ. Long COVID: Major findings, mechanisms and recommendations. Nat Rev Microbiol 2023;21:133–46. 36639608 10.1038/s41579-022-00846-2 PMC9839201

[26] Proal AD , VanElzakker MB. Long COVID or Post-acute Sequelae of COVID-19 (PASC): an overview of biological factors that may contribute to persistent symptoms. Front Microbiol 2021;12:698169. 34248921 10.3389/fmicb.2021.698169 PMC8260991

[27] Komaroff AL , Lipkin WI. Insights from ME/CFS may help unravel the pathogenesis of postacute COVID-19 syndrome. *Trends Mol Med* 2021;27:895–906. 10.1016/j.molmed.2021.06.002 PMC8180841 34175230

[28] WHO. Post COVID-19 Condition (Long COVID): Fact Sheet. Geneva: WHO, 2024.

[29] Chu L , Valencia IJ, Garvert DW et al Onset patterns and course of Myalgic Encephalomyelitis/Chronic Fatigue Syndrome. Front Pediatr 2019;7:12. 30805319 10.3389/fped.2019.00012 PMC6370741

[30] Al-Aly Z, Davis H, McCorkell L, et al. Long COVID science, research and policy. Nat Med 2024;30:2148–64. 10.1038/s41591-024-03173-6 39122965

[31] Chen C, Haupert SR, Zimmermann L et al. Global prevalence of post-coronavirus disease 2019 (COVID-19) condition or long COVID: A meta-analysis and systematic review. *J Infect Dis* 2022;226:1593–607. 10.1093/infdis/jiac136 PMC9047189 35429399

[32] Jason LA, Mirin AA. Updating the National Academy of Medicine ME/CFS prevalence and economic impact figures to account for population growth and inflation. *Fatigue Biomed Health Behav* 2021;9:9–13.

[33] Lim E-J, Ahn Y-C, Jang E-S et al. Systematic review and meta-analysis of the prevalence of chronic fatigue syndrome/myalgic encephalomyelitis (CFS/ME). *J Transl Med*. 2020;18:100. 10.1186/s12967-020-02269-0 PMC7038594 32093722

[34] Johnston S, Brenu EW, Staines D et al. The prevalence of chronic fatigue syndrome/ myalgic encephalomyelitis: a meta-analysis. *Clin Epidemiol* 2013;5:105–10. 10.2147/CLEP.S39876 PMC3616604 23576883

[35] Sunkersing D, Ramasawmy M, Alwan NA et al. What is current care for people with Long COVID in England? A qualitative interview study. BMJ Open 2024;14:e080967. 10.1136/bmjopen-2023-080967 PMC11107429 38760030

[36] WHO Regional Office for Europe. Service Delivery Models for People with Post COVID-19 Conditions. Copenhagen: WHO Europe, 2025.

[37] Wilson N, Beasley MJ, Pope C, et al. UK healthcare services for people with fibromyalgia: results from two web-based national surveys (the PACFiND study). *BMC Health Serv Res* 2022;22:989. 10.1186/s12913-022-08324-4 PMC9347075 35922796

[38] Johnson LB, Maloney EL. Access to care in Lyme disease: Clinician barriers to providing Care. *Healthcare* 2022;10:1882. 10.3390/healthcare10101882 PMC9601439 36292329

[39] Cairns V. Supporting patients with long-term problems after Lyme disease. BJGP Open 2020;4:bjgpopen20X101102. 10.3399/bjgpopen20X101102 PMC7465565 32546581

[40] Cantrell C, Reid C, Walker CS et al. Post-COVID postural orthostatic tachycardia syndrome (POTS): a new phenomenon. Front Neurol 2024;15:1297964. 10.3389/fneur.2024.1297964 PMC10998446 38585346

[41] AAPM&R. *Long COVID Compendium: Expert Strategies for Comprehensive Patient Care.* 2025. https://www.aapmr.org/members-publications/newsroom/member-news/2025/04/30/long-covid-compendium—expert-strategies-for-comprehensive-patient-care (April 2026, date last accessed).

[42] Sammito S , Thielmann B, Böckelmann I Update: Factors influencing heart rate variability — a narrative review. Front Physiol 2024;15:1430458. 39165281 10.3389/fphys.2024.1430458 PMC11333334

[43] Sundas A , Contreras I, Navarro-Otano J et al Heart rate variability over the decades: A scoping review. PeerJ 2025;13:e19347. 40321810 10.7717/peerj.19347 PMC12047215

[44] Shaffer F , Ginsberg JP. An overview of heart rate variability metrics and norms. Front Public Health 2017;5:258. 29034226 10.3389/fpubh.2017.00258 PMC5624990

[45] Goodship A, Preston R, Hicks JT et al NOT “Tang A, Aitken A”. *medRxiv* 2025.

[46] Sawyer A, Preston R, Leeming H et al. Wearable technology in the management of complex chronic illness: preliminary survey results on self-reported outcomes. Front Digit Health 2025;7:1662255. 10.3389/fdgth.2025.1662255 PMC12541780 41132394

[47] Clague-Baker N, Davenport TE, Wickens B et al. Pacing with a heart rate monitor for people with myalgic encephalomyelitis/chronic fatigue syndrome and long COVID: a feasibility study. Fatigue Biomed Health Behav 2025;14:1–23.

[48] Faghy M. *ERASE-LC: An Open-label, Clinical Feasibility Study of the Efficacy of Remdesivir for Long COVID*. 2025. https://clinicaltrials.gov/study/NCT05911906 (April 2026, date last accessed). 10.1186/s40814-026-01823-9 PMC13281550 42063202

[49] Lillie EO , Patay B, Diamant J et al The n-of-1 clinical trial: the ultimate strategy for individualizing medicine? Per Med 2011;8:161–73. 21695041 10.2217/pme.11.7 PMC3118090

[50] Visible. *The Founding 100*. 2022. https://www.makevisible.com/blog/the-founding-100 (April 2026, date last accessed).

[51] Visible. *Visible: What’s Happening in 2024*. 2024. https://www.makevisible.com/blog/visible-in-2024 (April 2026, date last accessed).

[52] Visible. *The Next Step: Our Beta Launch*. 2022. https://www.makevisible.com/blog/the-next-step (April 2026, date last accessed).

[53] Visible. Visible: *What’s Happening in 2025*. 2025. https://www.makevisible.com/blog/visible-whats-happening-in-2025 (April 2026, date last accessed).

[54] Visible. *Building Visible in Public*. 2022. https://www.makevisible.com/blog/building-in-public (April 2026, date last accessed).

[55] Wannheden C , Åberg-Wennerholm M, Dahlberg M et al Digital health technologies enabling partnerships in chronic care management: Scoping review. J Med Internet Res 2022;24:e38980. 35916720 10.2196/38980 PMC9379797

[56] Peeples M , Maity B. Digital health in chronic care and self-management. In: Kiel JM, Kim GR, Ball MJ (eds), *Healthcare Information Management Systems*. Cham: Springer, 2022, 209–22.

